# Digital health integration and technological advancements in Saudi Arabia’s healthcare system: a comparative analysis of clustered and non-clustered hospitals

**DOI:** 10.2478/abm-2026-0026

**Published:** 2026-08-31

**Authors:** Ahmed Abdullah Alshehri, Asaad Abdulrahman Abduljawad

**Affiliations:** Department of Health Care Management and Health Informatics, College of Health Sciences, Umm Al-Qura University, Mecca 11343, Saudi Arabia; Department of Public Health, College of Health Sciences, Umm Al-Qura University, Mecca 11343, Saudi Arabia

**Keywords:** digital health, electronic medical records, hospital clustering, telemedicine

## Abstract

**Background:**

Digital health is central to Saudi Arabia’s Vision 2030, with hospital clustering introduced to improve efficiency, interoperability, and patient outcomes. However, comparative evidence on digital health adoption between clustered and non-clustered hospitals remains limited.

**Objectives:**

This study evaluates the integration of electronic medical records (EMRs), telemedicine, and mobile health applications in clustered and non-clustered hospitals in Saudi Arabia, assessing their impact on patient-centered care and system performance.

**Methods:**

A convergent mixed-methods observational design was applied. Quantitative hospital-level data (2016–2024) from two clustered and two non-clustered hospitals were analyzed for EMR adoption, telemedicine use, patient satisfaction, and chronic disease management. Logistic regression and ARIMA models assessed adoption patterns. Qualitative data were obtained through semi-structured interviews with 53 participants (30 patients, 23 physicians) purposively sampled from an initial pool of 80; saturation was reached at the 30th patient and 23rd physician. Thematic analysis identified facilitators, barriers, and perceived benefits.

**Results:**

Clustered hospitals consistently outperformed non-clustered hospitals across all digital health indicators. By 2024, clustered hospitals achieved higher Patient-Centered Care Index (95.1% vs. 92.2%) and Chronic Disease Management Scores (96.1% vs. 92.8%). Logistic regression showed clustered hospitals had 1.8-fold higher odds of EMR adoption (95% confidence interval [CI]: 1.3–2.5) and 2.1-fold higher odds of telemedicine use (95% CI: 1.5–2.8). Qualitative findings revealed facilitators (system integration, leadership support), barriers (digital illiteracy, fragmented systems, limited training), and perceived benefits (reduced waiting times, improved follow-up, patient engagement).

**Conclusions:**

Clustering enhances digital health adoption by strengthening interoperability, standardization, and institutional support. Persistent challenges, particularly in non-clustered hospitals, highlight the need for targeted investment in infrastructure, workforce training, and digital literacy to ensure equity in achieving Vision 2030 goals.

Digital health technologies are transforming healthcare systems worldwide by enhancing patient safety, improving operational efficiency, and elevating the overall quality of care [1]. In Saudi Arabia, digital health adoption plays a central role in realizing Vision 2030, a national agenda aimed at modernizing and sustaining healthcare services through innovation [2]. Under the Health Sector Transformation Program (SHSTP), the Ministry of Health (MOH) has prioritized the implementation of electronic medical records (EMRs), telemedicine, and mobile health applications to improve service delivery across the country [3].

A major structural reform within this transformation is the reorganization of the healthcare system into integrated regional hospital clusters. These clusters are designed to enhance care coordination, promote standardization, and enable more efficient use of healthcare resources [4]. Clustered hospitals often benefit from prioritized access to digital infrastructure, including interoperable EMR systems and integrated telehealth platforms [5]. In contrast, non-clustered hospitals continue to face challenges such as fragmented systems, limited interoperability, and inconsistent adoption of digital tools [6].

Saudi Arabia’s healthcare system is centrally governed by the MOH, which manages the majority of public hospitals and leads national digital transformation strategies [7]. Before SHSTP, the system faced fragmented service delivery and limited interoperability. The clustering reform was introduced to unify governance, streamline management, and accelerate digital health integration nationwide.

Although digital health has gained considerable attention in Saudi Arabia, few studies have conducted direct comparisons between clustered and non-clustered hospitals with respect to digital health adoption and performance. Addressing this gap is essential to evaluating the effectiveness and equity of current health reforms, and to ensuring alignment with Vision 2030’s objectives of innovation, accessibility, and patient-centered care [8].

This study focuses on hospitals in Mecca and the Eastern Province, two regions selected for their strategic importance and diversity of healthcare environments. These areas offer a representative context for examining the nationwide implementation of digital health initiatives, with Mecca encompassing a mix of public and private hospitals, and the Eastern Province reflecting strong industrial and digital infrastructure capacity [9].

This study aims to comprehensively evaluate the integration of digital health technologies in Saudi Arabia, drawing on longitudinal hospital performance metrics and qualitative insights from key stakeholders. By focusing on digital platforms such as EMRs, telemedicine, and mobile health applications, the study seeks to generate evidence-based insights into how clustering influences the adoption and effectiveness of digital health solutions [10].

## Methods

### Study design

This study employed a convergent mixed methods design to evaluate digital health integration in Saudi Arabia [11]. The quantitative component analyzed hospital-level performance indicators from 2016 to 2024, while the qualitative component explored stakeholder perspectives through semi-structured interviews conducted in 2024. Findings from both components were integrated through triangulation.

The research was conducted in the Western region (Mecca city) and the Eastern Province, both selected for their demographic diversity and strategic relevance to the Saudi SHSTP. Four hospitals were included: two clustered and two non-clustered, each with comparable capacity (300–400 beds). Clustered hospitals were tertiary care centers, while non-clustered hospitals comprised secondary and tertiary facilities.

Ethical approval was obtained from the Research Ethics Committee of Swansea University (Ref: 280818) and the Institutional Review Board of Umm Al-Qura University (Ref: UQU-IRB-2024-05-221). Written informed consent was obtained from all participants before interviews. The study adhered to the principles of the Declaration of Helsinki. Hospital-level data were de-identified and used with institutional permission; no individual patient records were accessed.

### Quantitative methodology Data sources and variables

Hospital-level data were extracted from MOH databases and hospital information systems for the years 2016–2024.

Variables included:

- EMR adoption rates (%)
- Telemedicine consultations per 100 patients
- Mobile health application usage
- Patient-Centered Care Index (PCCI)
- Chronic Disease Management Score (CDMS)
- Staff training hours per clinician
- Patient satisfaction (survey-based mean scores)
- Follow-up compliance rates (%)
- Health outcome improvement measures (e.g., glycated hemoglobin A [HbA1c], hospital readmissions).

All metrics were aggregated at the hospital level and reported as annual averages from 2016 to 2024. No individual-level data were used. Data were cleaned and cross validated with internal MOH quality reports to ensure consistency and completeness. Access to MOH administrative datasets was granted through formal agreements and approved by both ethics committees; only de-identified hospital-level data were provided, and no individual patient records were accessed.

### Sampling and hospital selection

A purposive comparative sampling approach was used to ensure that hospitals represented both governance structures under the Saudi SHSTP. Four hospitals met the eligibility criteria: two clustered and two non-clustered facilities with comparable bed capacity (300–400 beds) and complete reporting of digital health indicators for the full 2016–2024 period.

Inclusion criteria for hospitals included:

- availability of continuous digital health reporting,
- classification as either clustered or non-clustered under SHSTP,
- comparable service scope and bed capacity.

Exclusion criteria included:

- incomplete annual reporting,
- hospitals undergoing restructuring that could distort performance trends.

The sample size of 4 hospitals is justified because the objective was comparative evaluation of two structural models rather than statistical generalization, and only a limited number of hospitals met all inclusion criteria across the 9 years.

A structured algorithm guided site selection: (1) list all hospitals in Mecca and the Eastern Province with complete 2016–2024 digital reporting; (2) classify as clustered or non-clustered under SHSTP; (3) apply comparability criteria (300–400 beds, similar service scope); (4) exclude facilities with incomplete data or ongoing restructuring. The final sample included two clustered tertiary hospitals (C1, C2) and two non-clustered secondary/tertiary hospitals (NC1, NC2).

### Composite indicator

Technology Utilization Score (TUS) was developed to capture overall adoption of digital tools:

TUS = (0.6 × EMR adoption rate) + (0.4 × telemedicine consultations per 100 patients). This composite measure was pilot-tested against other digital health metrics to ensure internal consistency.

The PCCI combined metrics such as care coordination, shared decision-making, and patient-provider communication. The CDMS reflected hospital-level chronic care indicators including HbA1c control rates, adherence to clinical pathways, and readmission avoidance. Both indicators followed MOH standard dashboards and were scaled from 0 to 100.

### Tool development and validation

All composite indicators were derived from MOH national dashboards. The TUS was pilot tested against other digital indicators for internal consistency and aligns with national digital maturity metrics [10]. The weighting structure (0.6 for EMR, 0.4 for telemedicine) reflects MOH prioritization benchmarks for core digital enablers. The PCCI is based on the MOH Patient Experience Program, which undergoes annual psychometric validation through national survey cycles and incorporates domains such as communication, coordination, shared decision-making, and continuity of care. The CDMS follows standardized MOH chronic care dashboards and is validated annually through clinical audits and benchmarking reports. All 3 indicators use a 0–100 scale to support consistent comparison across facilities and years.

### Rationale for analytical techniques

Analytical choices were guided by the structure of the dataset and the study objectives. Binary logistic regression was used to explore structural predictors of digital adoption because the outcomes represent institutional-level decisions (adopted/not adopted), and the results are interpreted descriptively given the small number of hospitals. ARIMA modelling was applied to the nine-year dataset as it is appropriate for assessing longitudinal trends in digital uptake across institutions. Covariates were included to adjust for structural differences rather than to infer causality, and together these analytical approaches provide exploratory insights while acknowledging limitations related to precision and generalizability.

### Qualitative methodology

Participants and sampling. A purposive sampling strategy was employed [12]. Eighty participants were recruited (40 patients, 40 physicians). Inclusion criteria required patients to have experience with EMRs, telemedicine, or mobile applications, while physicians were senior clinicians managing chronic diseases. Of these, 53 participants (30 patients, 23 physicians) were interviewed until saturation was reached.

### Data collection

Semi-structured interviews were conducted in Arabic during 2024 [13]. Interviews were audio-recorded with consent, transcribed verbatim, and translated into English. The interview guide, informed by prior literature and SHSTP policy, explored usability, accessibility, barriers, facilitators, and perceived impacts of digital health [14]. To ensure anonymity and systematic organization.

All participants were assigned unique identifiers. For senior physicians, IDs began with the prefix SP, followed by the hospital type (C1, C2, NC1, NC2), department acronym (e.g., Cardiology [CARD], Oncology [ONC], Endocrinology [END]), and a sequential number (e.g., Senior Physician from Clustered Hospital 1, Cardiology Department, first interviewee [SP-C1-CARD-01]). For patients, IDs consisted of the hospital type, department acronym, and sequence number (e.g., Patient from Clustered Hospital 1, Oncology Department, fourth interviewee [C1-ONC-04]). This coding system ensured confidentiality while enabling clear comparison between clustered and non-clustered hospitals and across specialties. Hospital-level quantitative data were obtained from MOH databases and internal hospital information systems for the years 2016–2024.

Data saturation was assessed continuously and reached when no new codes or perspectives emerged after consecutive interviews. Coding reliability was ensured through iterative coding, cross-checking of emerging themes, and maintaining an audit trail in NVivo to ensure transparency and replicability.

## Analysis

### Quantitative analysis

In addition to descriptive and group comparison analyses, a binary logistic regression model was conducted to assess the predictive value of hospital type (clustered vs. non-clustered) on two main outcomes: EMR adoption and telemedicine utilization. Independent variables included hospital type, bed capacity, regional location, and patient chronic disease prevalence. Results were reported as odds ratios (OR) with 95% confidence intervals (CI). Data were analyzed using R version 4.3.2 (R Foundation for Statistical Computing, Vienna, Austria) and IBM SPSS Statistics for Windows, version 26.0 (IBM Corp., Armonk, NY, USA). Descriptive statistics (mean, SD, median) were computed. Group comparisons were performed using independent t-tests, ANOVA, and Wilcoxon tests as appropriate. Effect sizes (Cohen’s d) and 95% CI were reported. ARIMA forecasting models were applied to examine adoption trends. Missing data (<10%) were addressed using multiple imputation by chained equations. Implausible values or outliers were identified through exploratory plots and were excluded if inconsistent with internal MOH data.

### Qualitative analysis

Thematic analysis followed Braun and Clarke’s 6-step framework [15]. Transcripts were coded inductively and deductively using NVivo 12 (QSR International).

### Integration of findings

Quantitative and qualitative findings were integrated through triangulation [16], enabling convergence, complementarity, and identification of discrepancies. Hospital performance metrics were compared with stakeholder perspectives to enhance interpretive validity. Quantitative and qualitative results were integrated through an explanatory triangulation approach, in which qualitative insights from patients and physicians were used to interpret and contextualize the 9-year performance trends observed in the quantitative analysis. Convergence was identified where both datasets highlighted stronger digital adoption, improved patient communication, and more coordinated workflows in clustered hospitals. Complementarity occurred when qualitative narratives explained the organizational and cultural mechanisms underlying quantitative differences, such as leadership alignment and digital readiness. No major discrepancies emerged; instead, qualitative findings enhanced the interpretability of temporal trends and clarified why adoption trajectories differed between hospital types.

## Results

### Quantitative findings

This study analyzed longitudinal hospital performance data from 2016 to 2024 across two clustered and two non-clustered hospitals in Saudi Arabia. **Table 1** presents institutional averages of key digital health and performance indicators during this period. Overall, clustered hospitals demonstrated consistently higher scores across all categories.

**Table 1.** Longitudinal performance metrics for clustered vs. non-clustered hospitals (2016–2024)

**Hospital type**	**Year**	**PCCI (%)**	**CDMS (%)**	**Staff training (h)**	**Patient satisfaction (%)**	**Follow-up compliance (%)**	**Health outcomes improvement (%)**	**Tech utilization score**
Non-clustered	2016	68.0	62.9	15.0	72.9	68.0	63.0	58.0
	2017	68.8	63.5	15.2	73.8	68.6	63.7	58.4
	2018	72.4	67.1	15.9	77.7	72.4	67.0	61.8
	2019	76.7	71.1	16.9	82.4	76.7	71.0	65.5
	2020	81.1	75.0	17.8	87.1	81.2	75.2	69.1
	2021	85.4	79.2	18.8	91.5	85.4	79.1	72.8
	2022	89.8	83.1	19.8	92.4	89.7	83.2	76.6
	2023	91.7	89.0	20.8	92.9	91.7	87.4	80.3
	2024	92.2	92.8	21.8	94.0	93.0	93.0	83.9

Clustered	2016	70.0	65.0	16.0	75.1	70.0	64.9	60.0
	2017	70.7	65.5	16.1	75.8	70.8	65.6	60.4
	2018	78.6	72.8	17.9	84.0	78.5	73.0	67.2
	2019	86.7	80.6	19.9	92.7	86.9	80.7	74.3
	2020	94.5	88.4	21.8	95.3	94.5	88.4	81.5
	2021	95.1	95.4	23.7	96.0	95.0	95.4	88.6
	2022	95.0	95.1	25.6	97.0	95.7	95.6	96.0
	2023	96.1	95.1	27.5	95.9	95.5	95.4	95.7
	2024	95.1	96.1	29.4	97.0	95.9	95.5	95.9

All data represent hospital-level averages derived from administrative datasets.

CDMS, Chronic Disease Management Score; PCCI, Patient-Centered Care Index; Tech Utilization Score, Composite of EMR and telemedicine adoption.

Between 2016 and 2024, the PCCI improved from 70.0% to 95.1% in clustered hospitals, compared to 68.0%–92.2% in non-clustered hospitals. The CDMS followed a similar pattern, increasing from 65.0% to 96.1% in clustered hospitals, and from 62.9% to 92.8% in non-clustered hospitals. These trends reflect enhanced coordination and adherence to chronic care protocols.

The TUS, a composite metric combining EMR and telemedicine adoption, rose from 60.0 to 95.9 in clustered hospitals, while increasing from 58.0 to 83.9 in non-clustered hospitals, indicating a steeper trajectory in digital uptake among clustered institutions.

Other performance metrics also showed consistent year-over-year gains. Patient satisfaction improved from 75.1% to 97.0% in clustered hospitals and from 72.9% to 94.0% in non-clustered hospitals. Staff training hours per clinician increased from 16.0 to 29.4 in clustered hospitals, compared to 15.0 to 21.8 in non-clustered ones, suggesting more intensive capacity-building efforts in the former. Follow-up compliance and health outcomes improvement both improved across the board, with clustered hospitals maintaining a consistent edge.

To assess statistical significance, binary logistic regression was used to examine the relationship between hospital type and key digital adoption outcomes. After controlling for covariates (age, gender, comorbidities), clustered hospitals had:

- 1.8 times higher odds of EMR adoption (95% CI: 1.3–2.5)
- 2.1 times higher odds of telemedicine utilization (95% CI: 1.5–2.8)

All variables represent hospital-level annual averages, not individual patient data. The percentages shown (e.g., 85% EMR adoption) reflect aggregate administrative records rather than interview responses. Group comparisons using independent *t*-tests and ANOVA were conducted for descriptive purposes, though statistical power was limited by the small sample (*n* = 4 hospitals). These results should therefore be interpreted as indicative, not inferential. The graph below illustrates the temporal trends in EMR and telemedicine adoption from 2016–2024, comparing clustered and non-clustered hospitals. The graph shows a clear divergence beginning in 2018, with clustered hospitals demonstrating faster and more consistent increases in digital adoption. In contrast, non-clustered hospitals displayed slower, irregular progression with periods of stagnation before 2021. These patterns align with SHSTP implementation phases and support the interpretation that structural integration accelerated digital readiness.

These trends reflect enhanced coordination and adherence to chronic care protocols. **Figure 1** illustrates the longitudinal trajectory of digital health adoption across clustered and non-clustered hospitals between 2016 and 2024. Clustered hospitals demonstrate a steeper and more consistent increase in digital utilization compared with non-clustered facilities.

**Figure 1.**
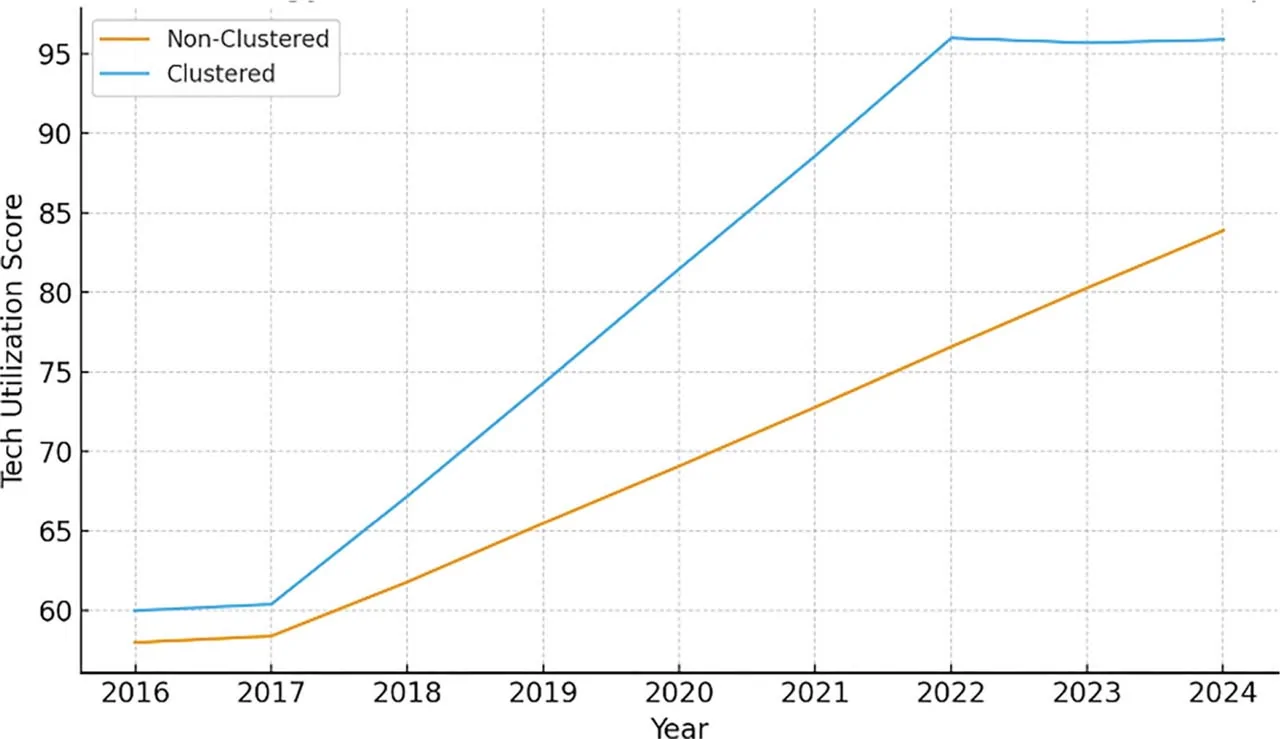
Temporal trends in digital adoption among clustered and non-clustered hospitals from 2016 to 2024. The TUS reflects combined EMR and telemedicine uptake. Clustered hospitals demonstrate a consistent and steeper upward trajectory, while non-clustered hospitals show slower and irregular adoption patterns. Values represent annual hospital-level averages. EMRs, electronic medical records; TUS, Technology Utilization Score.

Overall, the longitudinal data revealed a consistent pattern: clustered hospitals demonstrated earlier digital readiness, faster adoption of EMRs and telemedicine, and higher gains in patient-centered care and chronic disease management. These improvements were not isolated indicators but reflected broader organizational coherence and alignment with national digital transformation policies. The divergence between clustered and non-clustered hospitals widened after 2018, corresponding with the phased implementation of SHSTP reforms.

### Qualitative findings

Participants in clustered hospitals emphasized integrated systems and interoperability. One physician explained, “The cluster system makes referrals faster; patients move between hospitals without repeating tests” (SP-C1-CARD-02). A patient similarly noted, “It feels like one hospital even if I go to another branch” (C2-ONC-05).

By contrast, respondents in non-clustered hospitals reported frustrations with fragmented systems, inconsistent support, and weaker training. As one patient described, “Sometimes I have to carry my papers because the system in one hospital cannot see my records from another” (NC1-END-03). A physician added, “Technical support is not always available, so we often revert to manual processes” (SP-NC2-NEU-01).

Despite these barriers, both groups acknowledged the benefits of digital health. A clustered hospital patient reflected, “Before, I waited weeks for results; now, I get them in a day” (C1-CARD-07). Meanwhile, a non-clustered physician admitted, “Even with our challenges, telemedicine has helped reduce unnecessary hospital visits” (SP-NC1-ONC-04).

The qualitative themes emerging from patient and physician interviews across both hospital types are summarized in **Table 2**, highlighting key facilitators, barriers, and perceived benefits of digital health adoption.

**Table 2.** Themes from patients and physicians in clustered vs. non-clustered hospitals

**Theme**	**Clustered hospitals (patients)**	**Clustered hospitals (physicians)**	**Non-clustered hospitals (patients)**	**Non-clustered hospitals (physicians)**
Facilitators of Adoption	“I can access my lab results and prescriptions on the hospital app.” (C1-ONC-04)	“Our EMR is unified across facilities, which saves time.” (SP-C2-CARD-02)	“The app is there, but it’s hard to use without guidance.” (NC1-END-05)	“Training was limited, and staff adoption is uneven.” (SP-NC2-NEU-03)
Barriers to Implementation	“Older patients in my family don’t know how to use telemedicine.” (C2-CARD-07)	“Some colleagues resist digital systems; interoperability is partial.” (SP-C1-ONC-01)	“I face delays when the app crashes or does not load properly.” (NC2-ONC-06)	“Our systems don’t connect, so data gets lost between departments.” (SP-NC1-END-02)
Perceived Benefits	“Teleconsultations saved me from long hospital visits.” (C1-END-08)	“Digital tools reduced clinic crowding and improved chronic follow-up.” (SP-C2-NEU-04)	“I like reminders for appointments, but some-times they don’t arrive.” (NC1-CARD-03)	“Mobile apps help, but the lack of technical support reduces impact.” (SP-NC2-ONC-05)

## Discussion

This study demonstrates that digital health integration is a cornerstone of Saudi Arabia’s Vision 2030 healthcare reforms. Quantitative results showed that clustered hospitals consistently outperformed non-clustered hospitals in patient-centered care, chronic disease management, staff training, and overall technology utilization between 2016 and 2024. Qualitative insights triangulated these findings by highlighting that interoperability, leadership support, and streamlined workflows facilitated adoption in clustered hospitals, while fragmented systems, limited training, and digital illiteracy hindered progress in non-clustered facilities. Together, these findings provide robust evidence that clustering enhances both adoption and effectiveness of digital health interventions.

The performance gap between clustered and non-clustered hospitals reflects deeper organizational and governance differences embedded within the SHSTP. Clustered hospitals operate under unified governance structures that standardize decision-making, optimize resource allocation, and support coordinated procurement of digital platforms, technical support units, and cross-facility training [17].

Their interoperable EMR systems and integrated work-flows reduce duplication, accelerate referrals, and enable smoother transitions of care, advantages reinforced by prioritized investments in infrastructure such as fiber connectivity, cybersecurity, and digital maturity assessments. In contrast, non-clustered hospitals continue to rely on siloed systems, fragmented administrative arrangements, and inconsistent IT readiness, limiting data exchange and slowing digital adoption. These differences illustrate that performance outcomes are shaped not only by technology availability but also by organizational culture, leadership alignment, and systemic incentives inherent in the clustering model [18].

Additional organizational factors may also explain the performance gap observed between hospital types. Clustered hospitals benefit from stronger workforce digital readiness due to more structured training pathways, standardized competency requirements, and continuous exposure to system-wide digital initiatives. Financial incentives embedded within SHSTP reforms further prioritize digital maturity, encouraging clustered institutions to accelerate implementation and demonstrate measurable improvements. These organizational and policy mechanisms collectively reinforce the observed divergence and align with broader Vision 2030 objectives for integrated, digitally enabled healthcare delivery.

The advantages observed in clustered hospitals can be attributed to structural and organizational reforms embedded within the Saudi SHSTP. By integrating facilities under unified governance, clustered hospitals benefit from shared digital platforms, standardized clinical protocols, and economies of scale in workforce training [19, 20]. This contrasts with non-clustered hospitals, where siloed IT systems, fragmented care pathways, and bureaucratic inertia slow down digital adoption. These results reinforce the principle that clustering functions not only as an administrative strategy but as a digital enabler of health system transformation [21].

The temporal analysis also provides valuable insights. Adoption was steady between 2016 and 2019, accelerated sharply during the COVID-19 pandemic (2020–2022), and plateaued in 2023–2024 [22]. This trajectory is consistent with global evidence showing that pandemics accelerate uptake of telemedicine and remote monitoring [23]. However, sustaining momentum requires continuous investment, cultural adaptation, and workforce development to prevent stagnation.

Our findings align with national and international literature. Previous Saudi studies confirm that organizational reform and digital infrastructure are pivotal for transformation [24]. Globally, integrated models such as the UK’s NHS illustrate the importance of governance and stakeholder engagement in scaling EMR and telehealth solutions [25]. Estonia’s national electronic health record (EHR) system, supported by strong state-level policy and nationwide digital literacy campaigns, demonstrates how centralized, population-wide strategies can ensure consistent adoption of digital health across all facilities [26]. This mirrors the logic behind Saudi Arabia’s clustered model, in which integration under a unified governance framework accelerates digital transformation and minimizes disparities between institutions.

Similarly, Singapore’s integrated cluster-based healthcare system illustrates the benefits of linking hospitals through interoperable digital platforms. By embedding EMRs and telehealth into coordinated care pathways, Singapore has achieved measurable improvements in efficiency, patient safety, and chronic disease management [27]. These experiences highlight how clustering, when combined with digital enablers, creates system-wide gains, a finding echoed in our study, where clustered hospitals in Saudi Arabia outperformed non-clustered hospitals in both adoption and outcomes.

### Policy and practice implications

The results carry clear implications for policymakers. First, investment must be directed to non-clustered and rural hospitals to close adoption gaps, ensuring equity and consistency across the health system [28]. Second, targeted digital literacy campaigns for both patients and providers are essential to maximize the benefits of EMRs, telemedicine, and mobile health applications [29]. Third, embedding digital health training into routine clinical workflows and providing targeted incentives for staff participation could help reduce cultural resistance and normalize technology use [30].

Saudi Arabia’s heavy investment in artificial intelligence and predictive analytics presents an opportunity to build on current progress. Integrating these tools into clustered hospitals could drive efficiencies in chronic disease management, early detection, and personalized care [31]. However, careful governance and ethical frameworks will be needed to manage data security, algorithmic bias, and equitable access.

These findings directly align with the strategic objectives of Saudi Vision 2030 and the SHSTP, which emphasize interoperability, digital readiness, and patient-centered models of care. Clustered hospitals embody the Vision 2030 implementation roadmap by promoting integrated service delivery, unified health information systems, and enhanced accountability. The observed improvements in patient satisfaction, chronic disease management, and workflow efficiency reflect the expected outcomes articulated in national digital health strategies, including the National eHealth Strategy and the Digital Health Maturity Model adopted by the MOH [32].

Internationally, the performance trajectory of clustered hospitals mirrors successful large-scale digital transformation models such as the UK NHS integrated care systems, Estonia’s national EHR ecosystem, and Singapore’s cluster-based digital health architecture. These global examples reinforce the finding that digital transformation is most effective when embedded within coordinated governance structures, supported by workforce training, and aligned with broader policy mandates. By situating the study within both national and international frameworks, the results underscore that Saudi Arabia’s clustering model is consistent with global best practices in driving equitable and system-wide digital transformation [33]. While digital adoption contributed to performance differences, the clustering management structure, characterized by unified governance, shared infrastructure, and coordinated workflows, likely played an equally significant role. Thus, the observed outcomes reflect the combined influence of digital maturity and broader organizational reforms.

This study has several limitations. The quantitative data were derived from only 4 hospitals, limiting statistical power and generalizability. Although longitudinal, the dataset is institution-level rather than patient-level, restricting causal inference. Qualitative data were collected cross-sectionally in 2024, which limited insights into changes in perceptions over time. Additionally, geographic coverage was restricted to Mecca and the Eastern Province, underrepresenting rural and remote hospitals that may face distinct challenges. Despite these limitations, the mixed-methods approach strengthens validity by triangulating hospital records with stakeholder perspectives. Several limitations must be considered when interpreting these findings. First, the small sample of 4 hospitals limits statistical power and restricts the generalizability of model estimates, meaning the observed differences should be viewed as indicative rather than causal. Second, the use of composite indicators such as PCCI, CDMS, and TUS may introduce measurement constraints because these indices rely on administrative reporting cycles and may not capture all aspects of patient-centered care or digital maturity. Third, qualitative data were collected only in 2024, which may not fully reflect earlier phases of digital transformation and could amplify recent improvements in clustered hospitals. Finally, because clustered hospitals received earlier SHSTP investments, the data structure may inherently favor their performance trajectories, introducing a potential upward bias that should be acknowledged in interpretation.

Future studies should extend sampling to include rural and under-resourced hospitals, where digital equity issues may be most pronounced. Longitudinal qualitative designs would help capture evolving user experiences over time. Finally, further research should evaluate the impact of advanced technologies, such as AI, big data analytics, and predictive modeling, on the quality, safety, and efficiency of care in the Saudi context.

## Conclusion

This study provides one of the first longitudinal assessments comparing digital health adoption and patient-centered care performance between clustered and non-clustered hospitals in Saudi Arabia. By integrating nine years of administrative data with qualitative insights from patients and physicians, the study demonstrates that structural integration under the SHSTP may be strongly associated with faster digital readiness, higher patient experience scores, and more consistent improvements in chronic disease management. These findings highlight the importance of unified governance, coordinated investment, and workforce digital capacity in advancing national digital transformation objectives. While the results should be interpreted cautiously due to sample and measurement limitations, they offer actionable evidence for policymakers seeking to strengthen digital maturity across the health system and support equitable progress toward Vision 2030 goals.
